# High expression of antimicrobial peptides cathelicidin-BF in *Pichia pastoris* and verification of its activity

**DOI:** 10.3389/fmicb.2023.1153365

**Published:** 2023-06-09

**Authors:** Xufeng Dong, Hu Shan, Shubai Wang, Zhengjun Jiang, Shaojuan Wang, Zhihua Qin

**Affiliations:** ^1^College of Veterinary Medicine, Qingdao Agricultural University, Qingdao, Shandong, China; ^2^College of Animal Science and Technology, Qingdao Agricultural University, Qingdao, Shandong, China; ^3^Shandong Hwatson Biochem Co. Ltd, Weifang, Shandong, China

**Keywords:** antimicrobial peptides, eukaryotic expression, bacteriostatic effect, *in vitro* activity, *in vivo* activity

## Abstract

Antibacterial peptides are endogenous polypeptides produced by multicellular organisms to protect the host against pathogenic microbes, they show broad spectrum antimicrobial activities against various microorganisms and possess low propensity for developing resistance. The purpose of this study is to develop recombinant antibacterial peptide cathelicidin-BF by genetic engineering and protein engineering technology, and study its antibacterial activity *in vitro* and *in vivo*, so as to provide reference for the production and application of recombinant antibacterial peptide cathelicidin-BF. In this study, on account of *Pichia pastoris* eukaryotic expression system, we expressed and prepared antibacterial peptide cathelicidin-BF. Then, the minimum inhibitory concentration of antibacterial peptide cathelicidin-BF and the comparison with the antibacterial activity of antibiotics were determined through the antibacterial experiment *in vitro*. Chickens as infection model were used to verify the antibacterial peptide activity *in vivo*. The results show that the bacteriostatic ability of antibacterial peptide cathelicidin-BF is similar to that of antibiotics in certain concentration, and can reach the treatment level of antibiotics. Although the mode of administration of antibacterial peptide is still limited, this study can provide reference for the future research of antibacterial peptide.

## 1. Introduction

The prevention and treatment of bacterial diseases has long relied on traditional antibiotics such as penicillin. However, the range of bacterial pathogens that have developed antibiotic resistance is huge and new antibiotics are urgently needed. One alternative is the use of antimicrobial peptides that are derived from immune response products produced in response to invasion by bacteria and associated microbial factors (Hancock and Diamond, 2000; Hancock, 2001; Cunliffe and Mahida, 2004; Reddy et al., 2004; Hancock and Sahl, 2006; Takahashi et al., 2010; Wang, 2015). These peptides are an important part of animal immune defense systems and have the advantages of broad-spectrum antibacterial action and lack of bacterial resistance (Epand and Vogel, 1999; Scott et al., 2000; Lai and Gallo, 2009; Nguyen et al., 2011; Pasupuleti et al., 2011; Kang et al., 2019). Antimicrobial peptides have been used as feed additives to prevent and treat digestive tract inflammation such as diarrhea in livestock and aquatic organisms. These compounds have been shown to reduce or completely replace antibiotics for livestock rearing and reduce the incidence of disease. However, the use of antimicrobial peptides as clinical drugs has not been reported although they have demonstrated potent antibacterial activities *in situ* in mammalian hosts (Skerlavaj et al., 1996; Bulet, 1999; Hancock and Chapple, 1999; Hancock, 2000, 2001; Hunter et al., 2005).

Theoretically, antimicrobial peptides are ideal candidates for new antibiotics but their use has been hampered by a lack of experimental data of their action in animals. These data include stability, effectiveness and safety using *in vivo* models and this has limited their application. For example, polyphemusin I isolated from horseshoe crab has robust antibacterial activity *in vitro* but was not effective *in vivo* (Turner et al., 1998). Interestingly, sequence modification of the peptide enhanced its *in vivo* effectiveness but decreased its *in vitro* activity (Zhang et al., 2000). The primary factor that limits *in vivo* effectiveness of peptides may be their degradation by host proteases. Therefore, modifications such as liposome embedding, the use of precursors, the use of carrier proteins or sequence modification are key components of improving their effectiveness.

According to different classification standards, there are many ways to classify antimicrobial peptides, but it is generally believed that it is more meaningful to classify antimicrobial peptides according to their structural characteristics. Therefore, antimicrobial peptides are mainly divided into α- and β-Helix Collapse two types (Liu et al., 2015). Although antimicrobial peptides have potential in drug development, they still have some undesirable characteristics in clinical application, Natural peptides in gastrointestinal tract and other body fluids are usually unstable, resulting in low bioavailability of antimicrobial peptides in organisms (Cunliffe and Mahida, 2004; Tan et al., 2021).

cathelicidin-BF is a new antibacterial peptide isolated from *Bungarus multicinctus*, which is amphiphilic α-Helical conformation (Costa et al., 2019). cathelicidin-BF has broad-spectrum antibacterial activity, especially against gram-negative bacteria. It can be used as a good drug to treat acne vulgaris (Wang et al., 2008). Up to now, the research on cathelicidin-BF has mainly focused on the antibacterial effect, and cathelicidin-BF has rarely been reported on the immune regulation and epithelial barrier protection. Some re-searchers speculate that exogenous Cathericidin-BF may affect the development and healing of UC mucosal injury and inflammation (Wang et al., 2011). In addition, cathelicidin-BF shows low cytotoxicity and high stability to mammalian cells (Zhang et al., 2015).

In this study, we expressed the antimicrobial peptide cathelicidin-BF with the help of *Pichia pastoris* that were secreted into the culture medium and processed for use as a therapeutic drug. We examined *in vivo* antibacterial activity in chickens infected with a highly pathogenic *Escherichia coli* strain. Our data will provide a useful reference for further research into the use of antimicrobial peptides as clinical antibiotic reagents.

## 2. Results

### 2.1. Antimicrobial peptide gene splicing and *Pichia pastoris* expression

**Figure 1:**
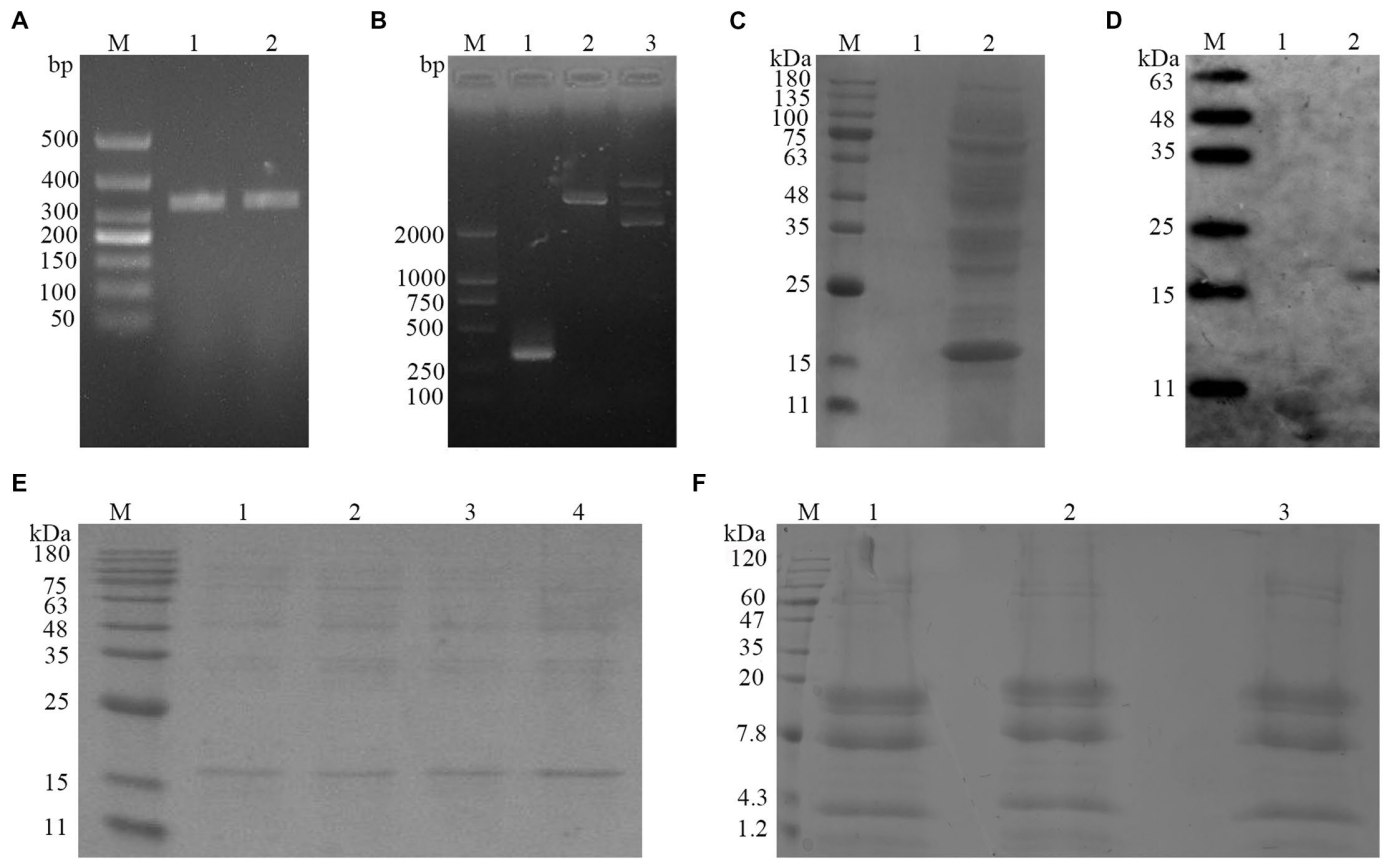
**(A)** Amplification of target gene: (Lane M) DL500 marker; (Lane 1–2) cathelicidin-BF gene fragment; **(B)** Identification of target gene: (Lane M) DL2000 marker; (Lane 1) PCR amplicon of the cathelicidin-BF gene; (Lane 2) *Eco*R I digestion of plasmid pPICα-cathelicidin-BF; (Lane 3) pPICZα-A digestion with *Eco*R I; **(C)** Expression of recombinant cathelicidin-BF in *Pichia pastoris*. Recombinant *Pichia pastoris* cells were continuously cultured and induced for 72 h by methanol, after centrifugation of the culture medium, the supernatant was used for SDS-PAGE protein electrophoresis: (Lane M) 180 kDa protein ladder; (Lane 1) control group: *Pichia pastoris* cells induced by methanol; (Lane 2) test group: the supernatant of culture medium where recombinant *Pichia pastoris* cells were cultured and induced by methanol. There was an obvious band between 15 and 25 kDa, and the band size is consistent with the expected size of the target protein; **(D)** Western blot: (Lane M) marker; (Lane 1) control group: *Pichia pastoris* cells induced by methanol; (Lane 2) test group: the supernatant of culture medium; **(E)** Purified product of target protein: (Lane M) 180 kDa protein ladder, (Lane 1–4) The eluted protein from the purification column; **(F)** The acidified compound antibacterial peptide was identified by Tricine-PAGE gel electrophoresis. (Lane 1 ~ 3) the acidified compound antibacterial peptide.

PCR amplification yielded a fragment of 310 bp that was consistent with the expected size of the cathelicidin-BF gene fragment. The cathelicidin-BF gene fragment was cloned into the *Pichia pastoris* expression vector and expressed in *Pichia pastoris*. Positive colonies were identified by *Eco*R I digestion and the insert identities were verified by DNA sequencing. The constructed expression plasmid pICZα-A-cathelicidin-BF was correctly inserted downstream of the Kex 2 cleavage site of the vector. Due to the insertion of the target fragment between the pPICZα-A poly-clonal sites, the original enzyme digestion site in the pPICZα-A multicloning site was destroyed so the plasmid could be identified by *Eco*R I digestion. Following *Eco*R I digestion, the electrophoresis patterns indicated the correct construct was cloned and was verified using sequence analysis. We induced expression of the cathelicidin-BF peptide using the methanol-regulated AOX promoter and examined culture supernatants for the presence of the peptide. The peptide migrating at 17 kDa was present only in the supernatant from the induced *Pichia pastoris* but not in parental cells or cells containing the empty vector or when methanol was excluded from the culture medium. Through repeated testing, we have found the optimal induction conditions for antibacterial peptides cathelicidin-BF. In BMGY medium, recombinant *Pichia pastoris* cells were inoculated with 1% volume. Then, at 200 rpm, pH = 5.5, and 22°C, methanol was added to the culture medium twice every 24 h, adding 1% of the total volume each time, with a phosphate concentration of 100 mM/L, and a ammonium sulfate concentration of 0.5%. After 240 h of induction, the expression of antibacterial peptides reached 0.5 g/L. We collected a large number of antimicrobial peptides cathelicidin-BF, concentrated the antimicrobial peptides by vacuum freeze-drying, and the treated antimicrobial peptides were stored in a low-temperature refrigerator. Because the antibacterial peptide cathelicidin-BF we prepared was a compound peptide expressed in series, we need to treat the compound peptide to turn the compound antibacterial peptide into a single active peptide. Under the action of acetic acid, the treatment results of composite antimicrobial peptides are as shown in the Figure 1F. The results were in line with our expectations. (Figure 1).

### 2.2. Minimal inhibitory concentration determination of antimicrobial peptide cathelicidin-BF

The MIC determination results of antibacterial peptide cathelicidin-BF are as Table 1.

**Table 1:** Minimal inhibitory concentration of antibacterial peptide cathelicidin-BF.

Tested strain	Antibacterial peptide cathelicidin-BF (μg/mL)
*Escherichia coli* (ATCC 25922)	27
*Escherichia coli* (E058)	29
*Escherichia coli* (Drug resistant bacteria)	29
*Staphylococcus aureus* (ATCC 29213)	30
*Staphylococcus aureus* (Drug resistant bacteria)	32

### 2.3. Determination of median lethal dose of test bacteria

The determination results of the median lethal dose of the test bacteria are as Tables 2, 3. The result is calculated according to the following formula:

$$\log{\mathrm{LD}}_{50}=\mathrm{Xk}-i\left(\sum p-0.5\right),$$

Xk in the formula represents the maximum logarithmic dose, i represents the difference between two adjacent logarithmic measurements, p represents the mortality of each dose group.

**Table 2:** Determination of median lethal dose of *Escherichia coli* to chicks.

Bacterial concentration (CFU/mL)	Number of samples	Number of deaths
1.00 × 10^7^	6	6
3.33 × 10^7^	6	6
1.11 × 10^6^	6	5
3.70 × 10^5^	6	4
1.23 × 10^5^	6	2
4.11 × 10^4^	6	1
1.37 × 10^4^ CFU/mL	6	0
**LD**_**50**_	**2.12 × 10**^**4**^ **CFU/mL**	

**Table 3:** Determination of median lethal dose of *Staphylococcus aureus* to chicks.

Bacterial concentration (CFU/mL)	Number of samples	Number of deaths
1.00 × 10^7^	6	6
3.33 × 10^7^	6	6
1.11 × 10^6^	6	4
3.70 × 10^5^	6	4
1.23 × 10^5^	6	3
4.11 × 10^4^	6	1
1.37 × 10^4^ CFU/mL	6	0
**LD**_**50**_	**2.27** × **10**^**5**^ **CFU/mL**	

In the chicken infection model, the dose used to inoculate the tested bacteria was twice the median lethal dose.

### 2.4. *In vitro* bacteriostatic test

**Figure 2:**
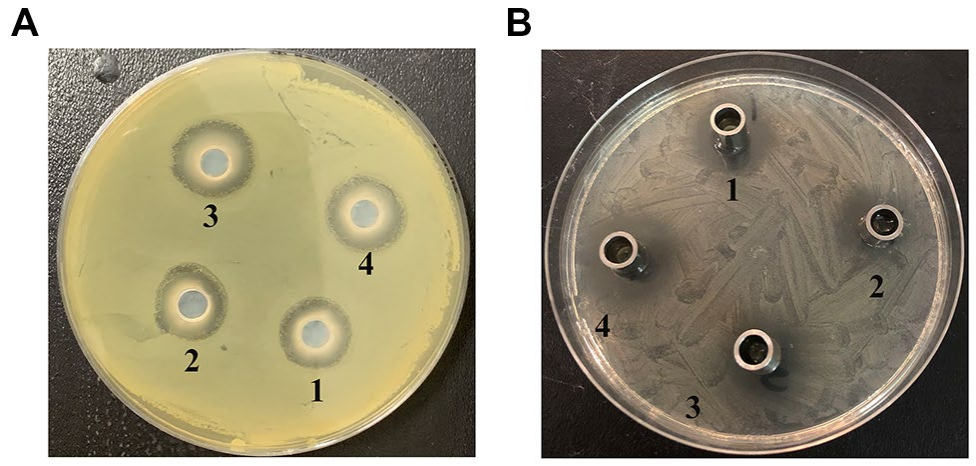
*In vitro* bacteriostatic test of standard strains. **(A)** *Staphylococcus aureus*: Amoxicillin (1); Streptomycin (2); Gentamicin (3); cathelicidin-BF (4); **(B)** *E. coli*: Ampicillin (1); Streptomycin (2); Amoxicillin (3); cathelicidin-BF (4).

**Figure 3:**
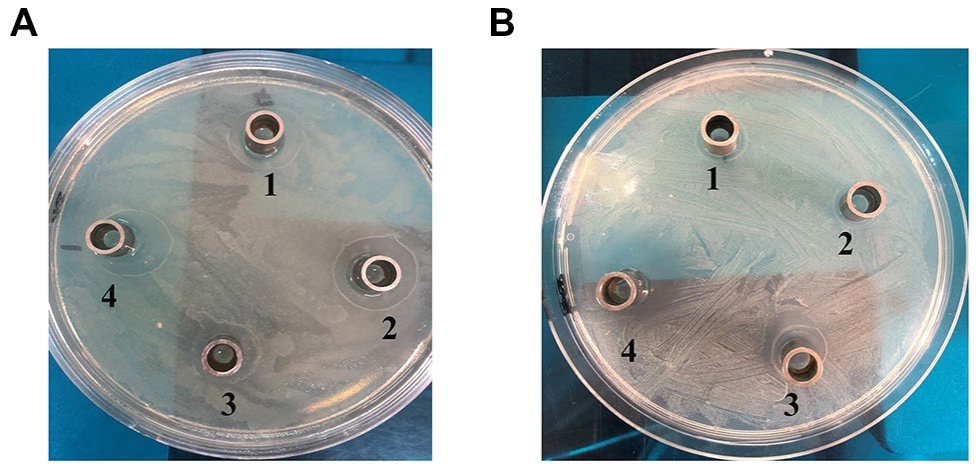
*In vitro* bacteriostatic test of drug resistant strains. **(A)** *E. coli*: Ampicillin (1); cathelicidin-BF (2); Amoxicillin (3); Gentamicin (4); **(B)** *S. aureus*: Ampicillin (1); Amoxicillin (2); Gentamicin (3); cathelicidin-BF (4).

**Figure 4:**
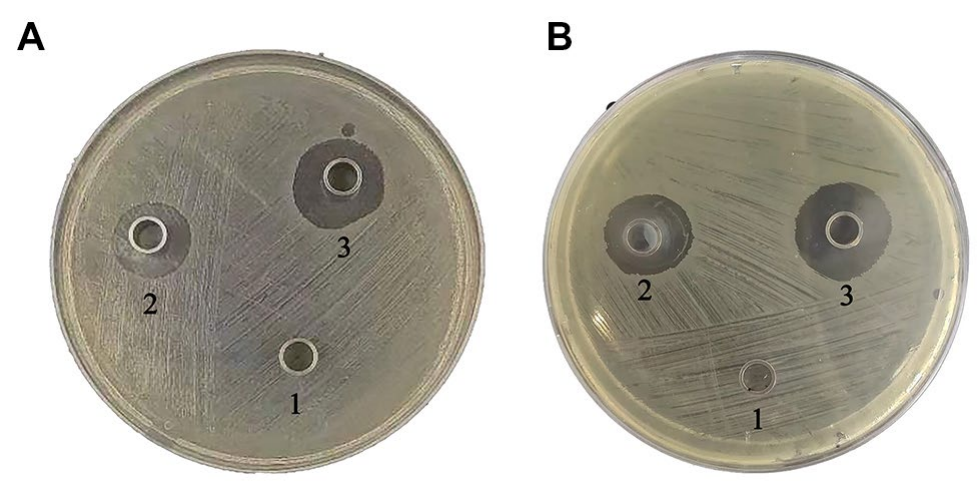
Comparison of *in vitro* antibacterial experiments. **(A)** *E. coli*: normal saline (1); secreted peptide (2); synthetic peptide (3); **(B)** *S. aureus*: normal saline (1); secreted peptide (2); synthetic peptide (3).

The secreted peptides were examined for its effects on bacterial growth *in vitro* using agar plate cultures (Figures 2, 3). Meanwhile, synthetic peptides were used to compare with secreted peptides (Figure 4).

The test results show that: according to the numerical order marked in the Figure 2A, the bacteriostatic circle of 10 μL (25 μg/mL) antibiotic solution were 24.6 mm, 26.9 mm, 27.5 mm. The bacteriostatic circle of 10 μL (30 μg/mL) antimicrobial peptide cathelicidin-BF solution was 26.8 mm. According to the numerical order marked in the Figure 2B, the bacteriostatic circle of 10 μL (25 μg/mL) antibiotic solution were 29.6 mm, 30.6 mm, 34 mm. The bacteriostatic circle of 10 μL (28 μg/mL) Antimicrobial peptide cathelicidin-BF solution was 30.9 mm.

The test results show that: the antibacterial effect of antimicrobial peptides was the same as that of Amoxicillin, and was better than that of Ampicillin and streptomycin, but not as good as that of Gentamicin.

The test results show that: according to the numerical order marked in the Figure 3A, the bacteriostatic circle of 10 μL (25 μg/mL) antibiotic solution were 16.5 mm, 17.3 mm, 30.5 mm. The bacteriostatic circle of 10 μL (29 μg/mL) antimicrobial peptide cathelicidin-BF solution was 31.2 mm. It can be seen from the bacteriostatic circle that the bacteriostatic effect of the antimicrobial peptide test group was significantly better than that of the antibiotic group, and there was little difference between the inhibitory effect of the antimicrobial peptide on drug-resistant bacteria and the standard strain at the same dose, which indicates that the antimicrobial peptide may have better resistance to bacterial drug resistance.

In Figure 3B, the bacteriostatic circle of 10 μL (25 μg/mL) antibiotic solution were 9.5, 10.2, and 20.5 mm. The bacteriostatic circle of 10 μL (32 μg/mL) antimicrobial peptide cathelicidin-BF solution was 27.5 mm. In addition, the bacteriostatic effect of Ampicillin and Gentamicin decreased sharply in the drug-resistant strain test, which indicates that the problem of bacterial drug resistance of Ampicillin and Gentamicin may be more prominent in actual production.

In Figure 4A, the bacteriostatic circle of 10 μL (30 μg/mL) synthetic peptides solution were 30 mm, the bacteriostatic circle of 10 μL (30 μg/mL) secreted peptides solution were 27 mm.

In Figure 4B, the bacteriostatic circle of 10 μL (30 μg/mL) synthetic peptides solution were 31.2 mm, the bacteriostatic circle of 10 μL (30 μg/mL) secreted peptides solution were 27.8 mm.

### 2.5. *In vivo* bacteriostatic test

**Figure 5:**
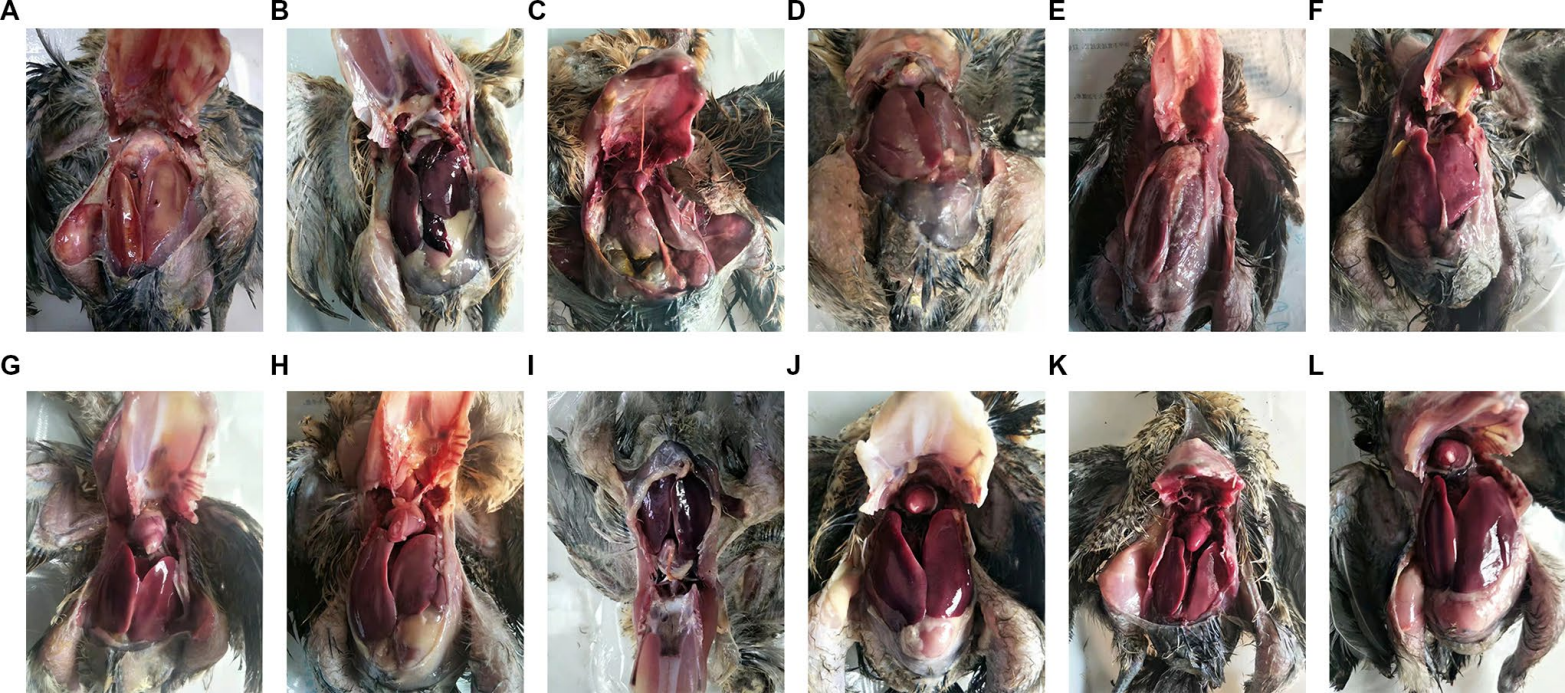
Animal Experiment. **(A – C)** Diagram of internal organs of chicken after death of *S. aureus* inoculation: the feathers of the chicken’s chest and abdomen fell off, and the skin presented purplish black swelling. After autopsy, the chickens were found to have significant subcutaneous congestion, accompanied by a large. Amount of gelatinous pink or yellowish red edema fluid the major signs included hepatomegaly, splenomegaly, and ascites, with pericardial effusion. The liver and spleen were purplish red; **(D –  F)** Diagram of chicken viscera after *E. coli* inoculation: The test chickens developed fibrinous pericarditis, with thickening of the pericardium, accompanied by pericardial effusion. The liver was significantly swollen with fibrous exudates on its surface. The spleen was congested and swollen，bile extravasation in some chickens, intestinal mucosa hyperemia or a little patchy hyperemia. **(G –  I)** Diagram of internal organs of experimental chickens in the treatment group: Compared to the untreated infected group, the chickens in the antibacterial peptide treatment group had normal viscera color and no significant pathological changes. **(J –  L)** Visceral organ diagram of control group: The color of each organ was normal without obvious lesions.

**Figure 6:**
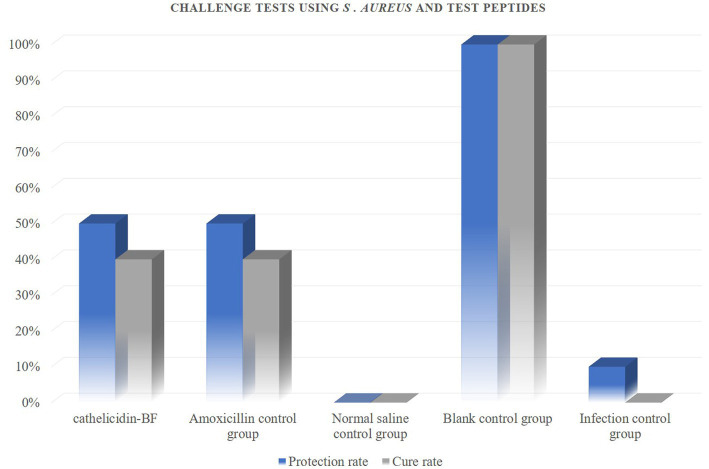
Challenge tests using *S. aureus* and test peptides. Antibacterial peptides cathelicidin-BF and amoxicillin have the same protective and therapeutic effects on chickens infected with *S. aureus*, with protection rates of 50% and treatment rates of 40%.

**Figure 7:**
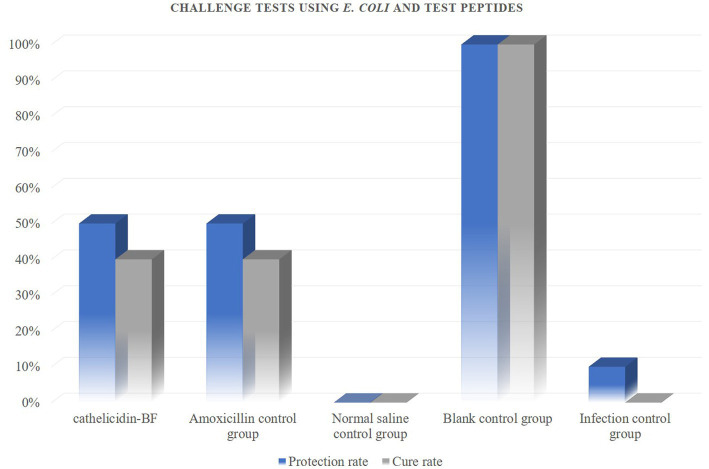
Challenge tests using *E. coli* and test peptides. Antibacterial peptides cathelicidin-BF and amoxicillin have the same protective and therapeutic effects on chickens infected with *E. coli*, with protection rates of 50% and treatment rates of 40%.

These peptides inhibited the growth of both Gram-positive and Gram-negative bacteria so we therefore examined whether this activity translated into *in vivo* activity. Our challenge bacterial strain for the chicken infection model was the pathogenic *S. aureus* and *E. coli* (Figures 5–7).

The results show that: administration of peptides reduced the severity of clinical symptoms and these animals gradually recovered compared with the infected control group.

## 3. Discussion

The rapid rise in the numbers of antibiotic resistant bacteria is also a concern of global food safety. Many antibiotics are no longer effective and this is a major concern for both animal and human health. Many countries have already adopted strict regulations for the use of antibiotics and the use of antibiotics as growth promoters in animals will certainly be globally banned. The animal feed industry is still using 20 types of antibiotics shared by human and livestock or as special additives for livestock and poultry. The entry of China into the World Trade Organization will seriously affect its global competitiveness in the international market if antibiotics are used as growth promoters. Therefore, the development of non-toxic, pollution-free, safe and efficient new antibacterial agents to replace antibiotics as feed additives has become a worldwide proposition. The research and development of antibacterial peptide biological agents can yield highly effective and safe new antimicrobials.

Other scholars used the *Bacillus subtilis* expression system and SUMO technology to isolate and purify the antibacterial cathelicidin-BF from the venom of the golden ring snake. The chimeric genes His-SUMO-cathelicidin-BF and His-SUMO proteolytic enzyme 1 were ligated to the vector pHT43 and expressed in *Bacillus subtilis* WB 800 N (He et al., 2015). This is the first report on the production of endotoxin free antibacterial peptide cathelicidin-BF by recombinant DNA technology, and also the first report on the production of highly active purified SUMO Protease 1 from *Bacillus subtilis*.

At present, there are few reports on *Pichia pastoris* expression of the antifungal peptide cathelicidin-BF. In this experiment, we selected the *Pichia pastoris* preferred codon to optimize the target gene. With the help of *Pichia pastoris* expression system, we obtained the antibacterial peptide cathelicidin-BF by methanol induction. After the fusion peptide was secreted into the culture medium, the target protein was purified by affinity chromatography (Luan et al., 2014; Karbalaei et al., 2020).

One of the innovations of this study is to optimize the conditions for the induction and culture of recombinant *Pichia pastoris*. Compared to typical induction conditions which is used to induce expression in recombinant Pichia pastoris (28 ~ 30°C, 220 ~ 250 rpm, pH = 6.0; Weidner et al., 2010; De Brabander et al., 2023; Turner et al., 2023), through repeated testing, we have found the optimal induction conditions for antibacterial peptides cathelicidin-BF. In BMGY medium, recombinant *Pichia pastoris* cells were inoculated with 1% volume. Then, at 200 rpm, pH = 5.5, and 22°C, methanol was added to the culture medium twice every 24 h, adding 1% of the total volume each time, with a phosphate concentration of 100 mM/L, and a ammonium sulfate concentration of 0.5%. After 240 h of induction, the expression of antibacterial peptides reached 0.5 g/L.

*In vitro* experiments, we compared the activity of synthetic peptides and secreted peptides, and the results were gratifying. Although secreted peptides were lower in purity than synthetic peptides, the difference in activity between the two was not significant under the same dose. However, the cost of synthesizing peptides is much higher than that of secreted peptides, this brings infinite possibilities for the application of secreted peptides in production.

*In vivo* experiments, it must be admitted that the number of sample animals may be one of the factors that restrict the test results. Increasing the number of test animals may be able to more accurately demonstrate the difference in the efficacy of antimicrobial peptides cathelicidin-BF and amoxicillin. In addition, although there were no significant differences in the activity status of chickens treated with the two drugs, after treatment with antimicrobial peptides, there were still residual infection symptoms on the organs of chickens. In the follow-up study, we will optimize the delivery method of antimicrobial peptides, hoping to achieve that antimicrobial peptide therapy can completely replace antibiotics.

Regarding the mode of action of antimicrobial peptides on test chickens, oral administration was one of the options, but due to the effect of the digestive enzyme and intestinal fluids in chickens, the effect was not ideal. Finally, intraperitoneal injection was selected as the drug delivery method. Some researchers had used nanomaterials to coat antimicrobial peptides, a smart epigallocatechin-3-gallate (EGCG)-loaded silk fibroin-based nanoparticle (NP) with the surface functionalization of antimicrobial peptides was constructed (Liu et al., 2022). This has brought us new ideas, and we have conducted new exploration based on it. In addition, compared to the method of prokaryotic expression (*E. coli*) (Tajbakhsh et al., 2018), the antibacterial peptide expressed by recombinant *Pichia pastoris* has a better antibacterial effect *in vitro*, which may be due to the modification of the peptide by the eukaryotic expression system.

The decomposition of intestinal fluid limits the therapeutic effect of antimicrobial peptides in animal intestinal infections. The application of antimicrobial peptides in animal intestinal therapy is currently relatively limited, and more attention has been focused on feed additives. Xie et al. (2020) explored effects of antibacterial peptide combinations on growth performance, intestinal health, and immune function of broiler chickens. In general, the two combinations of ABPs added in broiler’s basal diets can promote broiler growth and improve feed conversion rate instead of antibiotics. But in this study, antimicrobial peptides were added to the feed along with other additives, the intestinal fluid also had an impact on the decomposition of antimicrobial peptides and cannot be avoided. In another study, Xu et al. (2023) made an evaluation of the efficacy of the antimicrobial peptide HJH-3 in chickens infected with *Salmonella Pullorum*. The research results confirmed the role of antimicrobial peptides in the treatment of chicken intestinal bacterial infections. Similarly, the therapeutic effect of antimicrobial peptides was reduced by intestinal fluid. Regarding the use of AMPs, the choice of administration mode plays a very important role in the prevention and treatment of corresponding diseases (Knappe et al., 2019; Lee et al., 2019; Yu et al., 2020). At present, the combination therapy of nanomaterials and antimicrobial peptides may be full of vitality.

## 4. Materials and methods

### 4.1. Strains and plasmids

*Pichia pastoris* X-33 (His-Mut +) and pPICZα-A were purchased from Invitrogen (Carlsbad, CA, United States). *S. aureus* (ATCC 29213) and *E. coli* (ATCC 25922、E058) were from the clinical examination center of the Ministry of health of China. The clinical resistant strains of *E. coli* (drug resistance spectrum: AMP-STR-FLO-DOX-AMO-NEO) and *S. aureus* came from our laboratory (drug resistance spectrum: AMP-CIP-STR-ERY-FOX-GEN-TET). Synthetic peptides from Bankpeptide Biological Technology Co. Ltd. of China (purity ≥ 98%).

### 4.2. Reagents

*Xho* I, *Xba* I, *Sac* I and *Eco*R I endonucleases, T4 DNA ligase, r*Taq* and ex*Taq* enzymes were purchased from Takara (Japan). A DNA gel recovery kit was obtained from Shanghai Huashun Bioengineering (Shanghai, China). Zeocin and expression vector pPICZ α A and pierce micro BCA protein detection kit were purchased from Invitrogen. D-glucose, D-sorbitol, peptone, yeast extract and biotin were purchased from BBI (Kent, United Kingdom). Trichloroacetic acid, methanol, acetone and other reagents were of analytical grade and purchased from Shanghai Reagent No. 2 factory (Shanghai, China). Other reagents were of analytical grade.

### 4.3. Test drug and application method

According to the experimental method in this paper, the antibacterial peptides of cathelicidin-BF expressed by *Pichia pastoris* eukaryotic expression system were prepared. In agar diffusion test, amoxicillin was used as the control group to evaluate the antibacterial effect of antibacterial peptide cathelicidin-BF.

### 4.4. Culture media

Luria Bertani (LB), yeast extract, peptone and dextrose (YPD) medium and *Pichia pastoris* growth medium (BMGY) were used for bacterial and yeast cultivation as previously described (Pasupuleti et al., 2011). *Pichia pastoris* induction medium for plasmid-based expression utilized BMMY where glycerol was replaced with 0.5% methanol in BMGY.

### 4.5. Experimental animals

Sanhuang broiler chickens were purchased from Nanjing Sanyi Breeding factory and kept in isolation for 21 days prior to the start of the experiments. The normal growth and development of chicks was regarded as clinical health.

### 4.6. Antimicrobial peptide gene design and expression

The amino acid sequence of antibacterial peptide cathelicidin-BF was queried by NCBI as follows:

KFFRKLKKSVKKRAKEFFKKPRVIGVSIPF.

Because antimicrobial peptides are toxic in cells, in order to avoid cytotoxicity, we designed linker to connect the expression gene of antibacterial peptide, antibacterial peptides would be expressed in tandem. When the expressed antimicrobial peptides are collected, we add acetic acid to dissolve linker. Finally, we get an active antibacterial peptide. Three cathelicidin-BF were connected in series through linker (GTGDP).

The gene fragment was synthesized by Sangon Biotech (Shanghai, China) after modification. The synthesized target gene was used as a template for PCR amplification.

The PCR amplicons were digested with *Xho* I and *Xba* I and cloned into the *Pichia pastoris* expression vector pPICZα-A and introduced into *Pichia pastoris* competent cell by transformation using standard molecular biology methods. Colonies that grew on zeocin plates were purified and correct plasmids were identified using *EcoR* I digestion and PCR amplification using primers 5’AOX and 3’AOX. The insert identity was verified by DNA sequence analysis (Shanghai Boya, Shanghai, China). The plasmid pPICZα-cathelicidin-BF was linearized and introduced into *Pichia pastoris* by electroporation using the standard recommendations of the manufacturer (Invitrogen). Integration of the plasmid into the *Pichia pastoris* was determined using genomic DNA isolated using the liquid nitrogen freeze–thaw method. In addition, pPICZα-A empty carrier was tangentially transformed by enzyme and also entered into *Pichia pastoris* X-33 by means of electrotransformation, which was used as the control of follow-up work. The PCR cycling conditions were: 94°C 1 min and 30 cycles of 94°C 1 min, 56°C 1 min and 72°C 1 min and a final extension at 72°C for 10 min. Amplicons were analyzed using 1% agarose gel electrophoresis.

Positive recombinants were inoculated into YPD containing 100 μg/mL zeocin and cultured overnight at 30°C at 250 rpm and transferred to 100 mL BMGY medium with zeocin and incubated again until the OD_600_ nm reached 3 ~ 6. The cultures were centrifuged and the pellets were weighed and then suspended in 500 mL BMMY for induction and expression at different temperatures (20, 22, 24, 26, 28, and 30°C), speed (160, 180, 200, and 220 rpm) and times (12, 24, 36, 48, and 72 h). The culture supernatants were collected by centrifugation at 6,000 rpm for 10 min and WB test was used to verify the successful expression of antimicrobial peptides. Finally, we determined the anti-bacterial activity of the supernatant samples.

### 4.7. Antimicrobial peptide cathelicidin-BF sample treatment

Because the antibacterial peptide prepared was a complex protein in series, it has to be cleaved. Our method is acetic acid acidification. The 20% acetic acid solution was prepared and used for dissolving the sample (precipitation: 20% acetic acid = 1:4). The peptide dissolution conditions were 65°C and 2 ~ 4 h. The NaHCO_3_ solution was prepared and filtered for sterilization. The cracked samples were neutralized by NaHCO_3_ solution to pH 7.2 and centrifuged at 12,000 rpm at 4°C for 5 min. Because acidification treatment cannot completely cleave the antimicrobial peptide, passes through Pierce™ Protein Concentrators PES (3,000 MWCO, Thermo Fisher Scientific Inc.), in order to ensure the availability of antibacterial peptide cathelicidin-BF. The processed peptides were concentrated and stored in the refrigerator at − 20°C for standby.

### 4.8. Minimal inhibitory concentration determination of antimicrobial peptide cathelicidin-BF

Minimal inhibitory concentrations (MICs) were measured by standard microdilution method. The bacterial strains were incubated in nutrient broth at a concentration of 10^6^ CFU/mL containing appropriate aliquots of the peptides. MIC was defined as the lowest concentration of the peptides inhibiting visible growth after overnight incubation at 37°C. Each test was performed 3 times. The antibacterial peptide cathelicidin-BF diluted 2-fold dilution was added to the sterile 96 well plate, and 10 μL was added to each well from the first to the eleventh wells. The 12th hole was not added as the growth control. We added 100 μL of test bacterial suspension diluted by LB broth at 1:1,000 to each well, the 96 well culture plate was placed in a 37°C constant temperature incubator. We observed and recorded it every 2 h. At this time, the final concentration of antimicrobial peptides was 128, 64, 32, 16, 8, 4, 2, 1, 0.5, 0.25, and 0.125 μg/mL. Finally, the lowest concentration of antimicrobial peptides that completely inhibit bacterial growth in 96 well plates was identified as MIC.

### 4.9. *In vitro* bacteriostatic test

Antibacterial activity of the peptides was determined by a standard top agar assay. In brief, the bacterial strains used in this study were grown overnight in nutrient broth and inoculated into 10 mL of molten 1.0% nutrient agar at a final con centration of 10^6^ CFU/mL, followed by overlaying on a 90 mm Petri dish containing solidified 2% nutrient agar. After the top agar had solidified, 5-mm-diameter holes were punched out from each plate, and 10 μL of the peptide samples was added to the wells. The Petri dishes were incubated at 37°C. The peptide samples with antibacterial activity would form a clear zone around the holes. Meanwhile, synthetic peptides were used to compare with secreted peptides, synthetic and secreted peptides were added to the agar well (10 μL, 30 μg/mL).

### 4.10. Determination of median lethal dose of test bacteria

A single colony of pathogenic *S. aureus* and *E. coli* were cultured in LB at 37°C for 18 h and used to inoculate the chickens as previously described. We isolated bacteria from dead chickens and inoculated them into LB plate medium, selected single colonies with good growth and inoculated them in LB liquid medium, prepared the test bacterial solution, and measured their growth turbidity by plate counting method. We configured the cultured test bacteria into bacterial solutions of different concentrations, and the concentrations were set as 1.00 × 10^7^ CFU/mL, 3.33 × 10^7^ CFU/mL, 1.11 × 10^6^ CFU/mL, 3.70 × 10^5^ CFU/mL, 1.23 × 10^5^ CFU/mL, 4.1110^4^ CFU/mL, 1.37 × 10^4^ CFU/mL and 100 μL different concentrations of bacterial suspension were injected into the tested chickens through intraperitoneal injection, and the same amount of normal saline was used as the control group. The experimental chickens were randomly divided into two groups corresponding to *E. coli* and *S. aureus*. The concentration of each tested bacterial solution was verified with 6 chickens. During the experiment, the experimental chickens were raised in the most suitable environment for 7 days, and the growth and death were observed and recorded. The experimental chickens were dissected and observed after the experiment.

### 4.11. *In vivo* bacteriostatic test

Chickens (160) of the same weights were randomly divided into 2 parts, corresponding to two bacterial infection models of *S. aureus* and *E. coli*. The chickens were inoculated intraperitoneally with the tested bacteria, and the inoculation dose was twice the half lethal dose of the tested bacteria. Each part had five groups, with 10 chickens in each group, and received the following treatment (Table 4).

**Table 4:** Overview of experimental groups used for animal testing.

Group	Processing method	Administration route
cathelicidin-BF	Infection, inoculated intraperitoneally antimicrobial peptides	Inoculated intraperitoneally, 300 μg/d
Normal saline control group	Infection, inoculated intraperitoneally normal saline	Inoculated intraperitoneally, 1.5 mL/d
Amoxicillin control group	Infection, inoculated intraperitoneally amoxicillin	Inoculated intraperitoneally, 300 μg/d
Blank control group	Infection, No administration	
Infection control group	No infection, No administration	

Experimental animals were observed before and after bacterial inoculations and physiological indexes such as mental state, feeding, drinking, feces and respiration were monitored. Chickens that succumbed to bacterial infections were examined for pathological changes. The livers, spleens and mesenteric lymph nodes of these animals were isolated and homogenates were diluted with normal saline and cultured on LB plates for CFU counting. Animals that were challenged with bacterial cultures developed typical symptoms. These animals then received antimicrobial peptides daily for 5 days as outlined in Table 4. The animals were observed for clinical changes and animals that succumbed were subjected to pathological examinations and bacterial isolation as per above.

## 5. Conclusion

The results indicated that the cathelicidin-BF peptide produced in *Pichia pastoris* had a good therapeutic effect for chickens challenged with a pathogenic strain of *S. aureus* and *E. coli*. In particular, the peptide cathelicidin-BF had the best therapeutic effect that was equivalent to Amoxicillin treatment. The current study provides a reference method for the prevention and treatment of *S. aureus* and *E. coli* disease.

All financial, commercial or other relationships that might be perceived by the academic community as representing a potential conflict of interest must be disclosed. If no such relationship exists, authors will be asked to confirm the following statement.

## Data availability statement

The original contributions presented in the study are included in the article/supplementary material, further inquiries can be directed to the corresponding author.

## Ethics statement

The animal study was reviewed and approved by Ethics Committee of Qingdao Agricultural University. Written informed consent was obtained from the owners for the participation of their animals in this study.

## Author contributions

XD: methodology and writing—original draft. HS: project administration. SBW: technical support. ZJ and SJW: investigation. ZQ: supervision and review and editing. All authors contributed to the article and approved the submitted version.

## Funding

This research was supported by the Research and industrial development of new veterinary APIs and preparations, a major scientific and technological innovation project in Shandong Province (Major scientific and technological innovation projects in Shandong Province 2019JZZY020601); and the Study on the Mechanism of Immune Regulation of *Echinacea purpurea polysaccharides* on Macrophages of the Head Kidney of Turbot (General program of Shandong Natural Science Foundation, ZR2021MC173).

## Conflict of interest

ZJ and SJW are employed by Shandong Hwatson Biochem Co. Ltd.

The remaining authors declare that the research was conducted in the absence of any commercial or financial relationships that could be construed as a potential conflict of interest.

## Publisher’s note

All claims expressed in this article are solely those of the authors and do not necessarily represent those of their affiliated organizations, or those of the publisher, the editors and the reviewers. Any product that may be evaluated in this article, or claim that may be made by its manufacturer, is not guaranteed or endorsed by the publisher.
